# Association of β-casein gene polymorphism with milk production characteristics in Holstein cows

**DOI:** 10.3168/jdsc.2025-0746

**Published:** 2025-05-16

**Authors:** Danielle de C.M. da Fonseca, Alenia N. Vasconcellos, Elizangela D. Marino, Thais N. Chequer, Luisa M.F.S. Oliveira, João A. Negrão, Julio C. de C. Balieiro, Arlindo Saran Netto, Ana M.C. Vidal

**Affiliations:** 1 School of Animal Science and Food Engineering, University of São Paulo, Pirassununga, São Paulo, 13635-900, Brazil; 2 “Luiz de Queiroz” College of Agriculture, University of São Paulo, Piracicaba, São Paulo, 13418-900, Brazil; 3 School of Veterinary Medicine and Animal Science, University of São Paulo, Pirassununga, São Paulo, 13635-900, Brazil

## Abstract

The aim of this study was to analyze the association between the β-CN *CSN2* gene polymorphism, energy, enzymatic, and nitrogenous metabolites, and production, composition (fat, protein, lactose, total solids, nonfat solids), and SCC in the milk of Holstein cows submitted to the same nutritional and environmental conditions. Eighteen cows (6 A1A1 genotype, 6 A1A2 genotype, and 6 A2A2 genotype) were selected and matched for parity, calving season, and days in lactation. For the statistical analysis, a fixed linear model was applied in which genotypes were considered as a fixed effect and the dependent variables included metabolites, milk production, milk composition, and SCC. We found no statistically significant differences associating the genotype for the β-CN *CSN2* gene with milk production and composition characteristics or with energy, enzymatic, and nitrogen metabolites of blood and milk, except for variable glucose concentration (mg/dL), in which only the A2A2 genotype presented the highest means (67.78a) in relation to the other genotypes A1A1 (58.28b) and A1A2 (55.69b), where the letter a corresponds to the value of the highest mean and the letter b corresponds to the value of the lowest mean. Under the conditions in which the experiment was developed, the highest averages were numerically observed for variables milk production (kg/d), milk composition and yield (%), and urea nitrogen concentration (%) in the blood, milk, and urine of cows carrying the heterozygous genotype (A1A2) compared with both homozygous genotypes (A1A1 and A2A2), and we found that polymorphism of A1 and A2 alleles does not alter milk composition or blood and urinary metabolites.

## Highlights

• Information on cows from a dairy farm in Brazil was selected for the β-casein gene.
• No association was observed between β-casein gene polymorphism and milk production and composition.
• Polymorphism of A1 and A2 alleles does not alter milk composition and metabolites.

## Graphical Abstract

**Figure:**
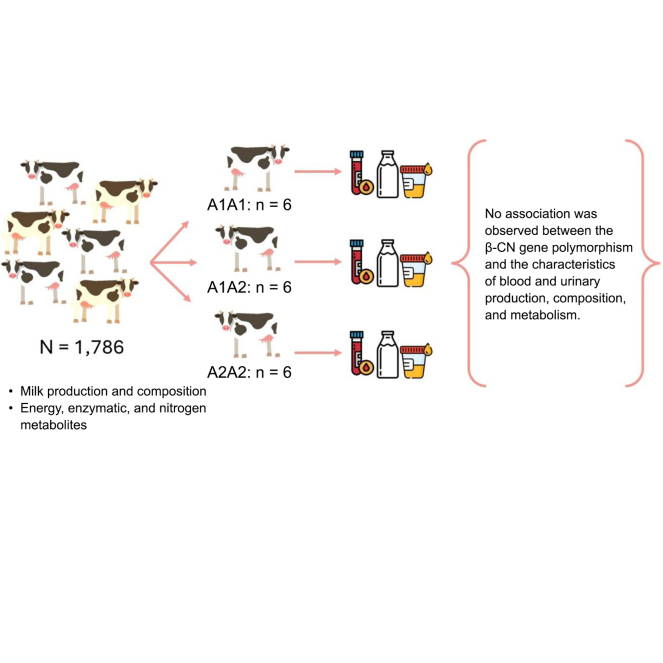
**Summary:** Information on milk production and composition was obtained from 1,786 lactating cows in a Brazilian commercial herd, for which data provided by the dairy farm in Brazil were adopted as selection criteria, such as genotypes for the β-casein gene extracted from the Clarifide Zoetis software, days in milk, and number of lactations. Thus, 18 cows were selected, analyzing the association between the genotype for the β-casein *CSN2* gene and milk production and composition characteristics and energy, enzymatic, and nitrogen metabolites of blood, milk, and urine. No association between β-casein gene polymorphism and any of the variables under study was observed.

Milk is one of the most ubiquitous and valuable agricultural commodities worldwide, with annual production of ∼930 million t in 2022, valued at $470 billion (FAO, 2024). Dairy trade flows can be substantially affected by changes in the trade policy environment (OECD-FAO, 2021), especially due to environmental concerns related to livestock-based food production, which generates nitrous oxide (N_2_O) and ammonia (NH_3_) emissions.

Nitrous oxide emissions result from the excretion of nitrogen in urine and feces during the nitrification and denitrification processes. Most nitrogen-rich compounds are excreted in urine and come from the complex nitrogen metabolism of ruminants, where CP is degraded by microorganisms into peptides, AA, and NH_3_ (Pečnik and Jevšinek Skok, 2024). Kohn et al. (2002) reported significant correlation between the CP level in the diet and the amount of nitrogen released into the environment. Thus, it is estimated that 75% to 85% of the excess protein consumed by a dairy cow is excreted in urine, feces, and milk (Kohn et al., 2002; Spek et al., 2013).

The MUN concentration is influenced by various factors, both nutritional and nonnutritional. Nutritional factors, particularly the quantity and composition of dietary proteins, play a significant role in the variation of MUN concentration (Barros et al., 2017; Wattiaux et al., 2019). Additionally, nonnutritional factors such as phenotypic and genotypic diversity including breed, parity, lactation days, milk production, milk quality, and milk components, also contribute to this variation (Arunvipas et al., 2003; Aguilar et al., 2012; Mangwe et al., 2025). Numerous studies have explored the differences in milk metabolites across various dairy species, feeding strategies, lactation stages, and other influencing factors (Caboni et al., 2019; Tomassini et al., 2019; Billa et al., 2020). However, there is a lack of information on changes in milk metabolites induced by bovine β-CN variants.

Beta-CN is composed of 209 AA and is controlled by a single gene located on chromosome 6, known as *CSN2* (Caroli et al., 2009). Within the *CSN2* gene, there are 13 genetic variants, with A1 and A2 being the most common, and A3, A4, B, C, D, E, F, H1, H2, I, and G being less common (Rahimi et al., 2015). The polymorphism of *CSN2* A1 and A2 alleles is genetically related to milk production results by a mutation occuring through natural selection that causes differences in AA residues. This variation arises from an SNP at codon 67 of exon 7 of the β-CN. In the A2 variant, the sequence CCT (cytosine-cytosine-thymine) encodes the AA proline, whereas in the A1 variant, the sequence is altered to CAT (cytosine-adenine-thymine), which encodes the AA histidine (Jaiswal and Sarsavan, 2014; Kay et al., 2021). This structural difference has been the focus of research (Brooke-Taylor et al., 2017; Asledottir et al., 2018; Nguyen et al., 2018), as this difference may influence the physicochemical and microstructural properties of milk (Elliott et al., 1999; McLachlan, 2001; Nguyen et al., 2018).

The objective of this study was to investigate the association between the β-CN gene polymorphism and various metabolites (energetic, enzymatic, and nitrogenous), as well as milk production and composition (including fat, protein, lactose, total solids, and nonfat solids) and SCC in Holstein cows that were kept under uniform nutritional and environmental conditions.

All experimental procedures applied in this study followed the ethical principles of animal experimentation, as well as the standards issued by the National Council for the Control of Animal Experimentation, which was approved by the Ethics Committee for the Use of Animals of the University of São Paulo Faculty of Animal Science and Food Engineering and registered under CEUA no. 9431111019.

Data were collected on the milk production and composition of 1,786 lactating cows, and the following data provided by the dairy farm in Brazil were adopted as selection criteria: genotypes for the β-CN gene extracted from the Zoetis Clarifide Plus software, days in lactation, and number of lactations. Thus, 6 cows were selected of each genotype (A1A1, A1A2, and A2A2), totaling 18 cows with ∼206 d of lactation, BW of 400 ± 50 kg, average BCS of 3.25 ± 0.25, and average milk production of 37.0 kg of milk/cow per day submitted to the same nutritional and environmental conditions.

Blood collection was performed according to the methodology described by Cooke et al. (2007); milk collection according to Vidal and Saran Netto (2018); and urine collection according to Ortolani (2003). To determine energy metabolites, the blood glucose concentration (mg/dL) was analyzed. For enzymatic metabolites, the concentrations of enzymes gamma-glutamyl transferase (**GGT**; U/L) and aspartate aminotransferase (**AST**; U/L) were analyzed. For nitrogen metabolites, concentrations of blood urea (**BU**; mg/dL), BUN (mg/dL), milk urea (**MU**; mg/dL), MUN (mg/dL), urinary nitrogen (**UN**; mg/dL), and urine urea nitrogen (**UUN**; mg/dL) were analyzed. Serum levels of energy, enzyme, and nitrogen metabolites were determined using the Mindray BS-120 chemistry analyzer (Mindray Bio-Medical Electronics Co. Ltd., China), with commercial kits from Labtest Diagnostic S.A. (Lagoa Santa, Minas Gerais, Brazil). Milk composition data (fat, protein, lactose, total solids, nonfat solids) and SCC values (cells/mL) were extracted from the report of results of analyses monthly performed on the property by the Milk Clinic (Piracicaba, São Paulo, Brazil) using the infrared device (composition) and flow cytometry (SCC).

To evaluate the association between β-CN gene polymorphism and milk production characteristics, the normality of residues was verified by the Shapiro–Wilk test, and the homogeneity between variances by the Levene test, adopting 5% significance level. For the mean test, the Tukey test was used, adopting 5% significance level. Data were analyzed by GLM procedure of SAS version 9.4 (SAS, 2016), adopting 5% significance level.

The association of the genotypes for the β-CN *CSN2* gene with milk production and composition characteristics is detailed in Table 1, and energy, enzymatic, and nitrogen metabolites in blood, milk, and urine are shown in Table 2.

**Table 1:** Association of the 3 different genotypes for the β-CN *CSN2* gene with the production and composition characteristics

Variable^1^	A1A1	A1A2	A2A2	SEM	*P*-value
MP (kg/d)	38.07	38.90	34.19	4.18	0.7025
Fat (%)	3.64	3.81	3.37	0.36	0.7019
Protein (%)	3.05	3.03	3.01	0.10	0.9642
Lactose (%)	4.60	4.75	4.79	0.08	0.2309
TS (%)	12.23	12.52	12.08	0.42	0.7589
NFS (%)	8.60	8.71	8.70	0.11	0.7242
Log_10_ SCC	1.65	1.58	1.65	0.16	0.9333
Fat yield (kg)	1.34	1.43	1.16	0.16	0.5037
Protein yield (kg)	1.13	1.18	1.03	0.12	0.6927
Lactose yield (kg)	1.75	1.84	1.64	0.19	0.7498

1 MP = milk production; NFS = nonfat solids content.

**Table 2:** Association of the 3 different genotypes for the β-CN *CSN2* gene with concentrations of energy, enzymatic, and nitrogen metabolites in blood, milk, and urine

Variable^1^	A1A1	A1A2	A2A2	SEM	*P*-value
Blood					
Glucose^2^ (mg/dL)	58.28b	55.69b	67.78a	1.62	0.0002
GGT (U/L)	44.21	39.73	45.28	3.62	0.5289
AST (U/L)	52.33	54.61	57.66	2.76	0.4135
BU (mg/dL)	36.54	40.09	34.94	2.61	0.3849
BUN (mg/dL)	17.08	18.73	16.33	1.22	0.3851
Milk					
MU (mg/dL)	34.91	43.90	37.20	3.15	0.1452
MUN (mg/dL)	16.31	20.51	17.39	1.47	0.1453
Urine					
UN (mg/dL)	915.13	1,039.27	1,065.34	127.92	0.6813
UUN (mg/dL)	427.43	485.64	497.83	59.82	0.6802

1 GGT = gamma-glutamyl transferase; AST = aspartate aminotransferase; BU = blood urea; MU = milk urea; UN = urine nitrogen; UUN = urine urea nitrogen.

2 Mean test in which the letter a corresponds to the value of the highest mean and the letter b corresponds to the value of the lowest mean.

For the blood glucose concentration (mg/dL) energy metabolite, a significant difference was observed (5% significance level) among the 3 genotypes, in which cows carrying the A2A2 genotype presented the highest averages (67.78a), followed by A1A1 (58.28b) and A1A2 (55.69b), where the letter a corresponds to the value of the highest mean and the letter b corresponds to the value of the lowest mean.

Although this variation is significant, it remains within the reference range for this species. On the other hand, no significant difference (*P* = 0.7025) was observed in milk production, with values of 38.07 kg/d for A1A1, 38.90 kg/d for A1A2, and 34.19 kg/d for A2A2. However, it is possible that the significant reduction in blood glucose concentration is related to the genotypes that numerically showed higher milk production.

Kaneko (2008) reported that the desirable blood glucose levels are between 45 and 75 mg/dL, thus demonstrating that despite this variation in this blood energy metabolite, the blood parameter is within the reference range, suggesting maintenance of homeostatic mechanisms in all genotypes.

Given the diversity of genotypes, we were interested in investigating possible differences in hepatic metabolism, which led to the analysis of the concentrations of GGT and AST enzymes. Additionally, other metabolites, such as BU, BUN, MU, MUN, UU, and UUN, are closely related to nitrogen metabolism. These metabolites can be influenced by nutritional and nonnutritional factors, including genotype, which is the main focus of this research (James et al., 1999; Cassel et al., 2005; Spek et al., 2013). However, under the experimental conditions of this study, when analyzing these parameters, no statistically significant differences were found between genotypes.

For the other enzymatic and nitrogen metabolites, no statistically significant differences were observed among the 3 genotypes at a 5% significance level. With the exception of GGT (mg/dL) and BUN (mg/dL) concentrations, all other metabolites observed are within the expected reference range, suggesting adequate metabolic activity. According to Kaneko (2008), in bovines, the desirable blood GGT levels are between 6.1 U/L and 17.4 U/L, and that of BUN is 15 mg/dL. González et al. (2006) explain that the increase in GGT concentration in dairy cows may be indicative of liver problems, such as hepatic lipidosis, *Fasciola hepatica*, and induction of increased glucocorticoid production. The high BUN concentration may be due to the high CP content of the diet because ∼70% of the protein is transformed into ammonia in the rumen and part is absorbed into the general circulation and through the bloodstream, reaching the liver and serving as material for urea formation, with urea levels having a direct relationship with the protein level in the diet (Wittwer, 2000).

For milk composition and SCC, no statistically significant differences were observed among the 3 genotypes at a 5% significance level. According to normative instruction No. 76 of the 2018 Brazilian legislation to ensure food safety and guarantee the quality of milk (Brasil, 2018), all composition (g/100g) and SCC (log cells/mL) milk parameters are within the recommended reference range, thus meeting the requirements of current Brazilian legislation.

Similar studies were carried out by Miluchová et al. (2023), who analyzed the effect of the *CSN2* gene genotypes on test-day milk yields in Slovak Holstein cows; Albarella et al. (2020), who analyzed the influence of the CN compound genotype on milk quality and properties; and Ivanković et al. (2021), who analyzed the genetic polymorphism and effect on milk production of the *CSN2* gene, with cows carrying the A2A2 genotype presenting 0.39%, 0.30%, and 0.76% more fat content and 0.31%, 0.48%, and 0.37% more protein content, respectively; and cows carrying the A1A1 genotype presenting 0.14%, 0.02%, and 0.57% more fat content and 0.22%, 0.16%, and 0.40% more protein content, respectively. In these studies, cows carrying the A1A2 genotype presented indifferent results, thus reinforcing that although previous studies found constituent levels higher than those observed in the present study, it was observed that the genotypes did not alter the physicochemical milk composition, and the quantitative difference observed between values for the different genotypes may be due to environmental conditions and external factors, as Ivanković et al. (2021) reported that the polymorphism of A1 and A2 alleles does not modify the milk composition.

Marino et al. (2024) analyzed the effect of *CSN2* gene A1 and A2 alleles on yield, composition traits, and SCC of milk from cows of contrasting genotypes in Brazil and observed that cows carrying the A2A2 genotype presented 0.13% and 0.07% more fat and protein content, respectively, and cows carrying the A1A1 genotype presented 0.09% less fat content and 0.01% more protein content. It should be highlighted that even when analyzing a large sample size, the quantitative differences observed in the different genotypes for the β-CN gene are not responsible for changes in the quality of milk from Holstein cows in tropical environments.

In summary, no statistically significant differences were found associating the genotype for the β-CN *CSN2* gene with the milk production and composition characteristics, nor with the energy, enzymatic, and nitrogen metabolites of blood and milk, except for variable glucose concentration (mg/dL), in which only the A2A2 genotype presented the highest averages. However, under the conditions in which the experiment was developed, a possible superiority of the heterozygous genotype (A1A2) in relation to both homozygous genotypes (A1A1 and A2A2) was numerically observed for variables milk production (kg/d), milk composition and yield (%), and UN concentration (%) in blood, milk, and urine, and we observed that the polymorphism of A1 and A2 alleles does not alter the milk composition or blood and urinary metabolites.
